# Transcription-Associated Metabolomic Analysis Reveals the Mechanism of Fruit Ripening during the Development of Chinese Bayberry

**DOI:** 10.3390/ijms25168654

**Published:** 2024-08-08

**Authors:** Li Sun, Shuwen Zhang, Zheping Yu, Xiliang Zheng, Senmiao Liang, Haiying Ren, Xingjiang Qi

**Affiliations:** 1 Institute of Horticulture, State Key Laboratory for Managing Biotic and Chemical Threats to Quality and Safety of Agro-Products, Zhejiang Academy of Agricultural Sciences, Hangzhou 310021, China; sunli@zaas.ac.cn (L.S.); zhangsw@zaas.ac.cn (S.Z.); yuzp@zaas.ac.cn (Z.Y.); zhengxl@zaas.ac.cn (X.Z.); liangsm@zaas.ac.cn (S.L.); renhy@zaas.ac.cn (H.R.); 2 Xianghu Laboratory, Hangzhou 311231, China

**Keywords:** Chinese bayberry, transcriptome, metabolome, fruit ripening, flavonoid

## Abstract

The ripening process of Chinese bayberries (*Myrica rubra*) is intricate, involving a multitude of molecular interactions. Here, we integrated transcriptomic and metabolomic analysis across three developmental stages of the *Myrica rubra* (*M. rubra*) to elucidate these processes. A differential gene expression analysis categorized the genes into four distinct groups based on their expression patterns. Gene ontology and pathway analyses highlighted processes such as cellular and metabolic processes, including protein and sucrose metabolism. A metabolomic analysis revealed significant variations in metabolite profiles, underscoring the dynamic interplay between genes and metabolites during ripening. Flavonoid biosynthesis and starch and sucrose metabolism were identified as key pathways, with specific genes and metabolites playing crucial roles. Our findings provide insights into the molecular mechanisms governing fruit ripening in *M. rubra* and offer potential targets for breeding strategies aimed at enhancing fruit quality.

## 1. Introduction

Fruits hold significance as an essential component of our daily dietary intake, providing vital nutrients for human health. Numerous fleshy fruits undergo ripening subsequent to the completion of the seed maturation process—a crucial developmental trait in fruit biology [1]. Fleshy fruits undergo alterations in sensory characteristics, including color, flavor, and texture, throughout the ripening process, marked by changes in numerous metabolites. The initial discernible changes associated with ripening involve modifications in fruit color, attributed to processes such as the accumulation of carotenoids and flavonoid pigments [2,3]. 

Flavonoids, recognized as vital nutritional and health components in fruits, have garnered widespread attention due to their remarkable antioxidant and anti-inflammatory capacities [4,5,6,7,8,9,10]. Compared to color, the content of flavonoids in fruit constitutes a more intricate trait and emerges as one of the most crucial quality attributes influencing consumer preference [11,12]. As a significant class of secondary metabolites prevalent in plants, flavonoids play crucial roles in plant growth and development [13]. The biosynthesis of flavonoids occurs via the phenylpropanoid pathway, where chalcone synthase and chalcone isomerase play pivotal roles as key enzymes, serving as limiting factors in this pathway [14]. Overexpression of the Petunia chi-a gene encoding chalcone isomerase in tomatoes resulted in a remarkable increase of up to 78-fold in fruit peel flavonols, highlighting the significance of chalcone isomerase in flavonol accumulation [15]. It has been reported that the expression of the dihydroflavonol 4-reductase gene exhibits two peaks during fruit ripening, indicating the significance of flavonoids in fruit development [16]. Quercetin, a subclass of flavonoids, plays a pivotal role in numerous plant physiological processes, including seed germination, pollen growth, and plant growth and development [17]. However, there is limited information available regarding the presence and role of flavonoids in *M. rubra*.

Sugars and organic acids are the predominant components of fruit, collectively contributing to the sweet and sour taste profile [18,19]. As fruits ripen, there is an accumulation of starch and sucrose, accompanied by another notable transformation observed in the ripening process of most fleshy fruits: the softening of texture [3], a significant factor influencing taste and shelf life [20]. Compelling evidence suggests that alterations in fruit texture result from structural modifications to cell wall components [21]. Studying the process of starch and sucrose accumulation during fruit development is instrumental in enhancing our understanding of quality formation during fruit ripening and guiding future efforts to regulate and improve fruit quality.

The *M. rubra*, indigenous to China, has been cultivated for millennia, not only in its native land but also in various other Asian countries. This subtropical fruit crop holds substantial economic importance and is extensively grown in the southern regions of China. Beyond being consumed fresh, the fruits of *M. rubra* undergo diverse processing methods, resulting in products such as juice, jam, confectionery, and wine. Its typical ripening period spans from May through June and into early July, particularly in key Chinese production regions, encompassing the Zhejiang, Hunan, Fujian, and Guangdong provinces [22]. The *M. rubra* fruit enjoys widespread popularity owing to its delectable taste and appealing color. Furthermore, it offers substantial nutritional and health benefits, given its richness in flavonoids and polyphenols [23].

The fruit of *M. rubra* is distinguished by its high concentration of cyanidin-3-O-glucoside (C3G), comprising at least 85% of its anthocyanin content. This concentration rivals that found in blackberries and notably surpasses levels observed in cranberries, blueberries, and raspberries [22], making *M. rubra* a notable source of natural antioxidant compounds [21]. Besides anthocyanins, other flavonoids present in the pulp contribute to anti-cancer, anti-diarrheal [24], and anti-bacterial effects. Currently, the main bayberry varieties are Dongkui and Biqi [23]. Studies on the ripening process of Biqi bayberries have revealed a transition in fruit color from green to dark red-purple, accompanied by a gradual increase in soluble solids and carbohydrates content, and a notable decrease in fruit firmness [25]. The soft texture exhibited by ripening *M. rubra* fruit makes it prone to mechanical damage during harvesting and transportation, adversely affecting its commercial value. In a previous study, we identified *MrBGALs* and investigated their expression patterns, suggesting their involvement in cell wall degradation [26]. However, the changes in the other quality indexes are still unclear. It is imperative to unravel factors influencing fruit flavor and nutrient composition during the development of bayberry fruit.

Advancements in sequencing technologies have paved the way for integrated analyses based on multi-omics, which have proven to be a valuable method for elucidating the regulatory mechanisms governing plant biological properties [18,27]. The integration of metabolome and transcriptome analysis allows for a comprehensive exploration of intricate changes in fruit development and maturation, covering the range from physiological phenotype to gene expression levels. In our prior research, we successfully identified all 15 *MrBGALs* in *M. rubra.* Subsequently, we performed a comprehensive analysis to explore the potential relationship between *MrBGALs* and the process of fruit ripening. This integrated analysis has been reported in numerous fruits [28,29,30]. In this study, the Dongkui variety was selected due to its widespread cultivation and economic significance. A combined transcriptome and metabolome analysis was employed to investigate the alterations in metabolic substances and gene expression in bayberry fruits at three distinct developmental stages. The emphasis was placed on understanding the accumulation of sugar, acid, and functional active products during the formation of fruit quality. Additionally, the potential regulatory mechanism underlying bayberry fruit softening was scrutinized. The findings from this research contribute significantly to establishing a crucial theoretical foundation for quality control and cultivation management in *M. rubra* production.

## 2. Results

### 2.1. Transcriptomic Analysis and Differentially Expressed Genes

To examine the genes that play crucial roles in fruit ripening, aiding the advancement of *M. rubra* breeding, we partitioned the fruit ripening process into three distinct developmental stages—unripe (UR), middle ripe (MR), and full ripe (FR)—of Dongkui, a major variety of *M. rubra*. This division allowed us to investigate the key genes contributing to the ripening process.

**Figure 1:**
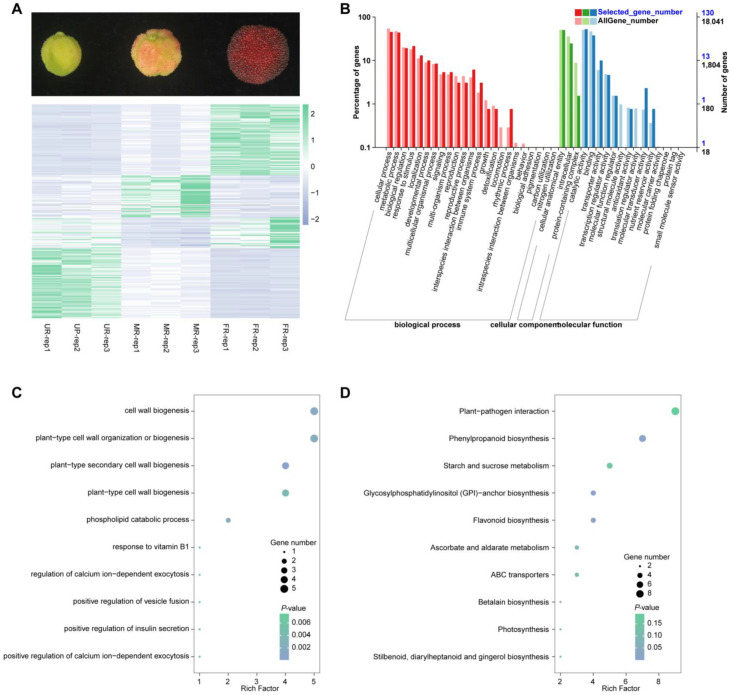
Differentially expressed genes during fruit ripening. (**A**). Representation of the three stages of *M. rubra* (UR, MR, and DR) and heatmap of the identification of four groups of key genes based on their expression patterns across the stages. (**B**). GO classification analysis of the identified genes. (**C**). GO enrichment analysis of the identified genes. (**D**). KEGG enrichment analysis of the identified genes.

Given the intricate nature of the ripening process in *M. rubra*, the impact of a single gene family may be limited. To address this complexity, we conducted an in-depth investigation, categorizing all differentially expressed genes across the three stages into four groups: those up-regulated in MR compared with UR, and FR compared with MR (up–up); those up-regulated in MR compared with UR, and down-regulated in FR compared with MR (up–down); those down-regulated in MR compared with UR, and up-regulated in FR compared with MR (down–up); and those down-regulated in MR compared with UR, and also down-regulated in FR compared with MR (down–down). These genes play roles throughout the entire ripening process and could provide clearer insights into the ripening process. Our analysis revealed 69 genes in the up–up group, 41 genes in the up–down group, 30 genes in the down–up group, and 68 genes in the down–down group (Figure 1A). In total, we successfully annotated 197 out of all the 208 candidate genes (Supplementary Table S2). To validate the reliability and accuracy of our RNA-seq results, we randomly selected three genes and performed a qPCR on RNA samples from the three different stages. The results demonstrated a significant correlation between the RNA-seq data and the qPCR findings (Supplementary Figure S1A,B).

We performed a gene ontology (GO) annotation for these genes and found that they are predominantly enriched in the following categories: cellular process, developmental process, molecular transducer activity, and nutrient reservoir activity (Figure 1B). Thus, we performed a GO enrichment analysis to uncover the functions of these genes. The results revealed that these genes were enriched in many biological processes involved in cell wall biogenesis; plant-type cell wall organization or biogenesis; and plant-type secondary cell wall biogenesis (Figure 1C). Fruit ripening, accompanied by softening, involves biochemical changes in cell wall fractions associated with the breakdown of cell wall polymers. These annotations suggest a strong correlation between these genes and fruit ripening and softening. A pathway analysis revealed that these genes were primarily enriched in plant–pathogen interaction, phenylpropanoid biosynthesis, and starch and sucrose metabolism (Figure 1D). These results imply that these genes may be pivotal in the accumulation of metabolites during fruit development.

### 2.2. Metabolome Analysis and Differential Metabolites

**Figure 2:**
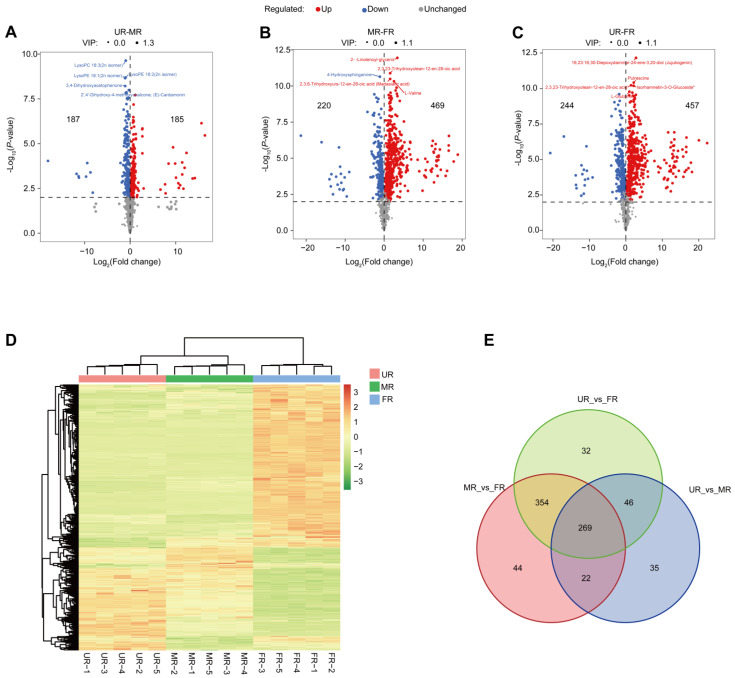
Differential abundance of metabolites during the fruit ripening. (**A**–**C**). Volcano plot illustrating the variation in metabolite abundance. (**A**): UR-MR, (**B**): MR-FR, and (**C**): UR-FR. (**D**). Heatmap displaying the relative abundance of identified metabolites across samples from different stages. (**E**). Differential abundance of metabolites identified in comparisons between stages (UR-MR, MR-FR, and UR-FR).

To explore metabolites from biological samples displaying significant biological and statistical variances during fruit development, we conducted a metabolome analysis on the LC-QTRAP platform in the three stages. In total, we identified 1071 predominant metabolites (Supplementary Tables S3 and S4). The PCA result revealed that samples from distinct stages could be categorized into three subgroups by PC1, PC2, and PC3, which accounted for 63.64%, 11.77%, and 2.90% of the variance, respectively (Supplementary Figure S2A). Samples from different stages cannot be classified into the same group, indicating that metabolites vary significantly between different stages. The correlation analysis results corroborated these findings (Supplementary Figure S2B). Additionally, the KEGG enrichment analysis revealed that these metabolites are significantly enriched in pathways such as the biosynthesis of plant secondary metabolites, ABC transporters, and flavonoid biosynthesis (Supplementary Figure S2C). Interestingly, according to the LipidMaps database, these metabolites are predominantly enriched in fatty acids and conjugates, as well as flavonoids (Supplementary Figure S2D). Subsequently, we conducted a differential analysis between stages. Comparing the UR stage to the MR stage, we identified 185 up-regulated metabolites and 187 down-regulated metabolites (Figure 2A). Comparing the MR stage to the FR stage, we found 469 up-regulated metabolites and 220 down-regulated metabolites (Figure 2B). Lastly, comparing the UR stage to the FR stage, we identified 457 up-regulated metabolites and 244 down-regulated metabolites (Figure 2C).

The results of a hierarchical clustering of metabolite profiles from different samples revealed that samples from the same stage were largely clustered into one subclass. Moreover, there were significant differences in the relative abundance of metabolites between different stages (Figure 2D). To explore the differential abundance of metabolites between different stages, we utilized the VIP scores obtained from the results of the OPLS-DA, a multivariate analysis model. By integrating univariate analysis *p*-values and fold change, we refined the selection of differential metabolites. We identified 269 metabolites that were differentially expressed in all three groups (Figure 2E, Supplementary Table S5). Among these 269 metabolites, flavonoids (49 out of 269) and lipids (49 out of 269) emerged as the two predominant metabolite classes.

**Figure 3:**
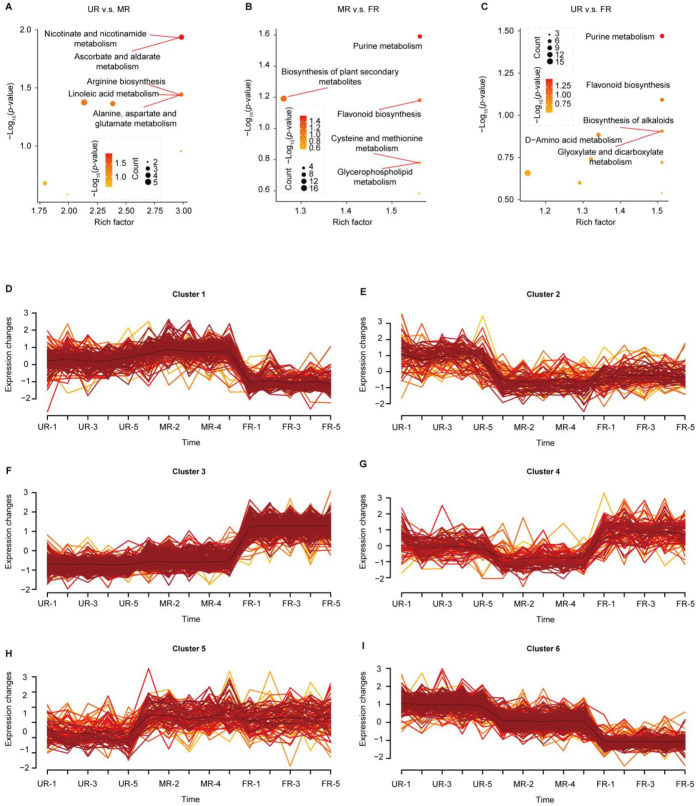
KEGG pathway enrichment analysis of differentially abundant metabolites and time-course profiling of metabolites. (**A**–**C**). KEGG pathway enrichment analysis of differentially abundant metabolites between stages (UR-MR, MR-FR, and UR-FR). (**D**–**I**). Metabolite clusters identified through time-course profiling.

To elucidate the functional implications of the distinct metabolites, we conducted a KEGG analysis. This analysis unveiled that metabolites differing between the UR and MR stages are notably enriched in ascorbate and aaldarate metabolism, nicotinate and nicotinamide metabolism, and arginine biosynthesis pathways (Figure 3A). During the MR-FR transition in post-fruit development, these differing metabolites exhibit an enrichment in purine metabolism, the biosynthesis of plant secondary metabolites, and flavonoid biosynthesis pathways (Figure 3B). Furthermore, metabolites differing between the UR-FR stages also demonstrate enrichment in purine metabolism and flavonoid biosynthesis (Figure 3C). Importantly, these distinct metabolite functions exhibit a discernible correlation with flavonoid biosynthesis.

### 2.3. Time Course of Metabolome

To comprehensively understand the temporal dynamics of metabolic changes occurring during different developmental stages, we conducted a timing analysis from the UR to the FR stage. All metabolites were classified into six clusters. Within clusters 1, 2, 3, and 5, flavonoids emerged as the predominant metabolites, constituting 35 (24%), 19 (22%), 88 (19%), and 28 (29%), respectively. Conversely, lipids emerged as the dominant metabolites in cluster 4, representing 25 (25%) of the total. In cluster 6, amino acids and derivatives emerged as the principal metabolites, accounting for 31 (18%) (Supplementary Figure S3). During the UR to MR stage, clusters 2, 4, 5, and 6 exhibited significant changes. Among these clusters, clusters 2, 4, and 6 showed reductions, whereas cluster 5 displayed an increase. Conversely, during the MR to FR stage, clusters 1, 3, 4, and 6 experienced notable changes. Specifically, clusters 1 and 6 demonstrated reductions, while clusters 3 and 4 showed increases (Figure 3D–I). The co-expression changes of these metabolites accompany the maturation process of development. Consequently, their functions are interlinked throughout the entire fruit development process. Therefore, we conducted a KEGG analysis for each cluster. In cluster 1, we observed an enrichment of metabolites associated with glycolysis/gluconeogenesis (Supplementary Figure S4A). Glycolysis involves the breakdown of glucose for energy generation, facilitating diverse biochemical and physiological activities crucial for fruit ripening. Conversely, gluconeogenesis entails the synthesis of glucose or other sugar molecules from non-sugar substrates, ensuring the maintenance of the requisite energy levels and carbon sources during fruit maturation. These processes synergistically promote fruit development and ripening, while supplying essential energy and carbon substrates. The metabolites in cluster 1 exhibit a reduction post-fruit development, suggesting a potential decline in metabolic activity or a shift in metabolic priorities. Cluster 2 displays an enrichment of metabolites associated with the biosynthesis of plant hormones and secondary metabolites (Supplementary Figure S4B). Furthermore, other clusters exhibit enrichment in pathways such as aminoacyl-tRNA biosynthesis, tropane, piperidine, and pyridine alkaloid biosynthesis, olfactory transduction, and bile secretion (Supplementary Figure S4C–F). Notably, clusters 5 and 6 are enriched in flavonoid biosynthesis, and metabolites within both clusters undergo changes throughout fruit development, indicating the involvement of flavonoids in this process.

### 2.4. Conjoint Analysis of Transcriptome and Metabolome Data

**Figure 4:**
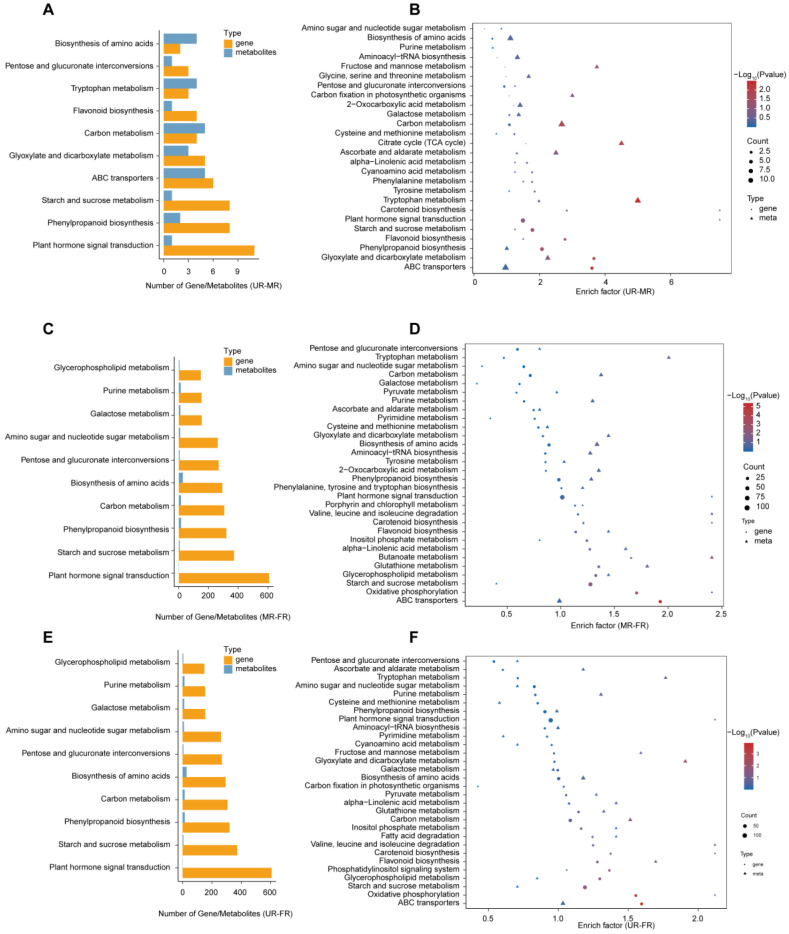
KEGG annotation and enrichment of identified genes and metabolites in this experiment. The highest number of the identified genes and metabolites, (**A**)**:** UR-MR, (**C**): MR-FR, (**E**): UR-FR. The KEGG enrichment of the identified genes and metabolites, (**B**): UR-MR, (**D**):MR-FR, (**F**): UR-FR.

To delve deeper into the identification of the key genes and metabolites influencing fruit ripening and softening, we employed the Two-Way Orthogonal Partial Least Squares (O2PLS) method to explore the correlation between gene expression and metabolites during the three stages. The joint score analysis between different stages indicated that, based solely on the joint score, the transcriptome and metabolome datasets could not be distinguished from each other. This suggests a similarity between different stages in terms of their transcriptomic and metabolomic profiles (Supplementary Figure S5A–C). This observation implies that there may be overlapping or coordinated changes in gene expression and metabolite levels across different stages of fruit development, highlighting potential interactions and regulatory networks between the transcriptome and metabolome during fruit ripening. The top 15 genes with the greatest influence on the formation of metabolites are displayed according to the order of the loading scores (Supplementary Figure S5D–F, Supplementary Tables S6–S8). The PCA result indicated that samples from distinct stages could not be categorized into the same group, signifying differences between these stages. Additionally, both the transcriptome and metabolome data from the same stage consistently clustered together, suggesting coherence and consistency between the transcriptomic and metabolic profiles within each stage (Supplementary Figure S5G–I). Furthermore, we identified the top 10 KEGG pathways with the highest number of identified genes and metabolites. Compared to the UR stage, there are 26 common KEGG pathways in both gene and metabolite enrichment in the MR stage. Meanwhile, there are 64 and 68 common KEGG pathways in the FR-to-MR and FR-to-UR stages, respectively (Supplementary Figure S5J–L). Our analysis revealed that, compared to the UR stage, genes and metabolites in the MR stage predominantly participate in pathways such as plant hormone signal transduction, phenylpropanoid biosynthesis, starch and sucrose metabolism, and flavonoid biosynthesis (Figure 4A). The KEGG enrichment analysis further demonstrated that these genes and metabolites are enriched in pathways including ABC transporters, glyoxylate and dicarboxylate metabolism, phenylpropanoid biosynthesis, flavonoid biosynthesis, and starch and sucrose metabolism, among others (Figure 4B). Similarly, compared with the MR stage, genes and metabolites are enriched in similar pathways in the FR stage (Figure 4C,D). Moreover, compared with the UR stage, the number of genes and metabolites is the same in the MR-FR stage, but the KEGG enrichment differs (Figure 4E,F). Interestingly, starch and sucrose metabolism and flavonoid biosynthesis are consistently ranked higher in all three groups.

### 2.5. Flavonoid Biosynthesis Changes during Fruit Ripening

**Figure 5:**
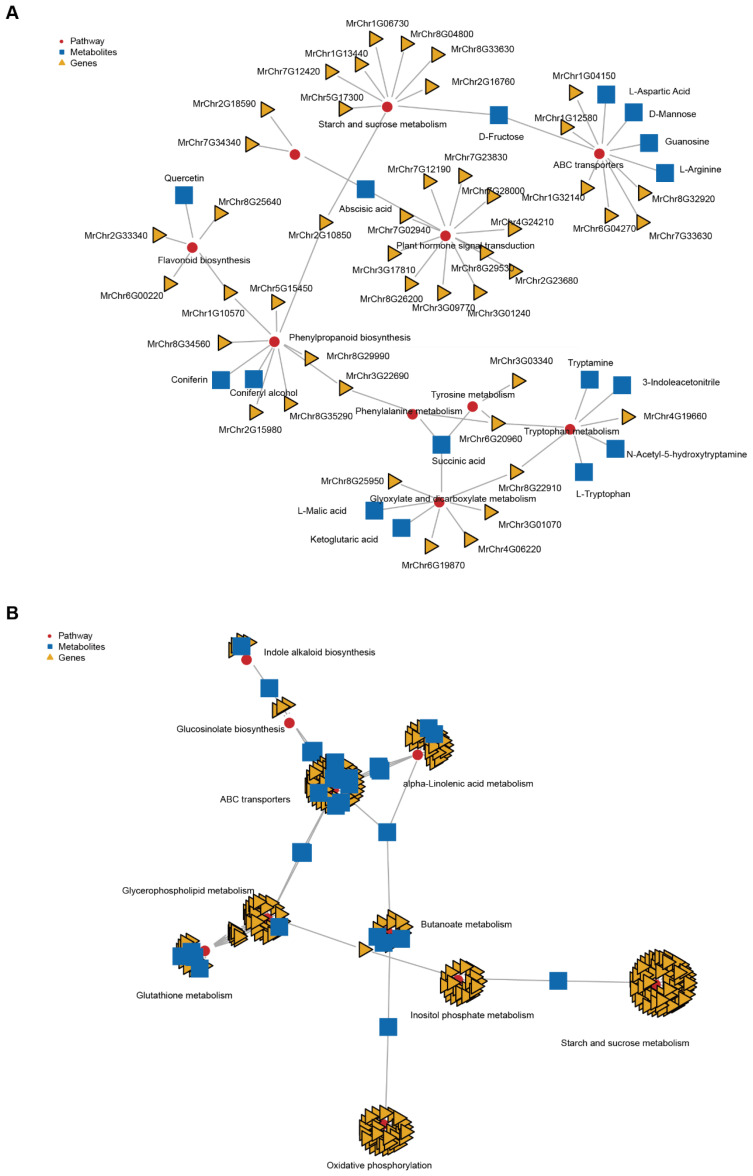
The relationship between differential pathway groups, genes, and metabolites by conjoint analysis ((**A**): UR-MR; (**B**): MR-FR). UR-FR exhibits notable similarities with MR-FR.

In comparison to the UR stage, we have identified four correlated differentially expressed genes and one differentially abundant metabolite related to flavonoid biosynthesis by the conjoint analysis of the transcriptome and metabolome in the MR stage. These genes are *MrChr1G10570*, *MrChr2G33340*, *MrChr6G00220*, and *MrChr8G25640*, along with quercetin (Figure 5A, Supplementary Table S9). Notably, *MrChr6G00220*, encoding a chalcone synthase gene, shows down-regulation, while *MrChr1G10570*, encoding a trans-cinnamate 4-monooxygenase gene; *MrChr8G25640*, encoding bifunctional dihydroflavonol 4-reductase; and *MrChr2G33340*, encoding vacuolar-sorting receptor 6, which is categorized as the chalcone isomerase in KEGG annotation, exhibit up-regulation. Among these genes, *MrChr6G00220* and *MrChr2G33340* represent limiting factors in flavonoid biosynthesis. Additionally, the abundance of quercetin is increased. Quercetin is a potent antioxidant, which may effectively prevent fruit softening during the ripening process. Comparatively, when comparing the MR stage to the FR stage, we found 23 differentially expressed genes and 3 metabolites (Figure 5B, Supplementary Table S10). Interestingly, both *MrChr1G10570* and *MrChr2G33340* are still up-regulated, and the abundance of quercetin continues to increase. Thus, during the fruit ripening process, *MrChr1G10570* and *MrChr2G33340* continue to affect flavonoid biosynthesis, alongside the continued accumulation of quercetin. 

### 2.6. Starch and Sucrose Metabolism Changes during Fruit Ripening

In comparison to the UR stage, we have identified eight differentially expressed genes and one differentially abundant metabolite associated with starch and sucrose metabolism by the conjoint analysis of the transcriptome and metabolome, all of which are up-regulated in the MR stage (Figure 5A, Supplementary Table S11). Subsequently, in comparison to the MR stage, we have identified 80 differentially expressed genes and one differentially abundant metabolite related to starch and sucrose metabolism in the FR stage, with 62 genes showing up-regulation (Figure 5B, Supplementary Table S12). Notably, three genes, *MrChr1G13440*, *MrChr2G16760*, and *MrChr8G33630*, exhibit up-regulation in both the UR-MR and MR-FR stages. Specifically, *MrChr1G13440* encodes L-type lectin domain-containing receptor kinase SIT2, which is categorized as alpha-amylase in KEGG annotation; *MrChr2G16760* encodes a disease resistance protein RUN1, which is categorized as the sucrose synthase in KEGG annotation; and *MrChr8G33630* encodes a probable sucrose-phosphate synthase 1, which is categorized as the sucrose-phosphate synthase in KEGG annotation. These genes are key components of the sucrose accumulation process. Additionally, D-Fructose, one of the most abundant sugars in nature, showed increased levels in UR-MR, and the abundance of D-Glucose 6-phosphate increased in the MR-FR stage, which could be attributed to the up-regulation of the above genes.

## 3. Discussion

*M. rubra* indeed offer a delectable and healthful option for one’s diet. Their richness in secondary metabolites, particularly flavonoid compounds, contributes significantly to their nutritional value and potential health benefits. In this study, our findings shed light on the intricate molecular mechanisms underlying the ripening process of *M. rubra*. With the ripening period being brief and coinciding with the plum rain season, understanding the genetic and metabolic factors governing this process is imperative for mitigating rapid softening and reducing economic losses. Through a comprehensive analysis integrating transcriptomic and metabolomic data, we identified the key genes and metabolites associated with fruit ripening, with a particular focus on the Dongkui, a major subspecies of *M. rubra*.

Our investigation involved the partitioning of the ripening process into three distinct developmental stages: UR, MR, and FR. By categorizing differentially expressed genes into four groups based on their expression patterns across these stages, we elucidate the dynamic changes occurring during ripening. Gene ontology annotation revealed an enrichment in processes such as cellular and metabolic processes, suggesting the involvement of these genes in fruit ripening, including cell wall modification and metabolism. Furthermore, pathway analysis highlighted the significant involvement of genes in protein metabolism, sucrose metabolism, and various secondary metabolite pathways, emphasizing the importance of metabolites during fruit development. The subsequent metabolome analysis provided further insights into the biochemical changes accompanying fruit ripening, with significant variations observed in metabolite profiles across different stages. Our findings align with previous studies on *M. rubra*, which also identified a substantial role for flavonoids and other secondary metabolites in fruit ripening and quality. For instance, similar research has highlighted significant concentrations of anthocyanins, proanthocyanidins, and primary metabolites during the ripening process, underscoring the importance of these compounds in determining fruit characteristics [31]. In our study, flavonoids and lipids emerged as predominant classes among the identified metabolites during the fruit ripening process. Given the significant functions of flavonoids, many previous studies have reported genes involved in flavonoid biosynthesis, especially those related to anthocyanin synthesis pathways [32,33]. However, our study goes beyond anthocyanin synthesis, to highlight other differentially expressed flavonoid-related metabolites and their corresponding differentially expressed genes. Moreover, our timing analysis of metabolome data revealed distinct temporal patterns in metabolite abundance, reflecting coordinated metabolic changes during fruit development. The observed reductions in certain metabolites during the post-development stages suggest potential shifts in metabolic activity or metabolic priorities, highlighting the dynamic nature of fruit ripening processes. 

The conjoint analysis of transcriptomic and metabolomic data through O2PLS revealed a strong correlation between gene expression and metabolite profiles, reinforcing the interplay between these molecular components during fruit ripening. Through the integration of transcriptomic and metabolomic data, we identified key genes and metabolites influencing fruit ripening and softening, with pathways such as plant hormone signal transduction, phenylpropanoid biosynthesis, and starch and sucrose metabolism prominently involved. Sugars play a crucial role in fruit development, significantly determining fruit flavor. Previous studies have highlighted the importance of sucrose phosphate synthase (SPS) in the synthesis of sugar compounds in various fruits. SPS genes are up-regulated during the development and ripening of Chinese bayberry, linking SPS expression with changes in carbohydrate metabolism and fruit quality. Similarly, higher expression levels of SPS genes (*MrSPS1*, *MrSPS2*, *MrSPS3*) are associated with increased soluble sugar content during bayberry ripening. These findings are consistent with our results, where we identified differentially expressed SPS genes closely related to sucrose metabolism [34,35].

Specifically, our analysis highlighted changes in flavonoid biosynthesis and starch and sucrose metabolism during fruit ripening. Differentially expressed genes related to flavonoid biosynthesis, such as chalcone synthase and chalcone isomerase, alongside the accumulation of quercetin, underscored the importance of flavonoids in fruit ripening. Similarly, the up-regulation of genes associated with starch and sucrose metabolism, including alpha-amylase and sucrose synthase, coincided with the accumulation of sugars such as D-fructose during ripening. The findings on flavonoid biosynthesis during fruit ripening, particularly the differential expression of genes such as *MrChr1G10570*, *MrChr2G33340*, *MrChr6G00220*, and *MrChr8G25640*, along with the increased abundance of quercetin, have significant implications for breeding strategies. Notably, *MrChr8G25640*, encoding dihydroflavonol 4-reductase, has been previously reported to be involved in anthocyanin synthesis in Chinese bayberry fruit, with its expression increasing during fruit development [36]. The up-regulation of *MrChr1G10570* and *MrChr2G33340*, which continue to affect flavonoid biosynthesis through the fruit ripening stages, presents opportunities for targeted breeding approaches. These genes can be utilized to develop molecular markers for desirable fruit quality traits, such as enhanced antioxidant properties and improved fruit firmness, which are crucial for extending shelf life and reducing post-harvest losses. Furthermore, the identification of quercetin as a key metabolite with potential to prevent fruit softening suggests that breeding programs can focus on increasing quercetin levels to improve fruit quality. Additionally, these insights can be applied to optimize post-harvest handling and storage practices, by targeting the metabolic pathways involved in flavonoid biosynthesis, thereby maintaining fruit quality and extending marketability.

## 4. Material and Methods

### 4.1. Plant Material

The *M. rubra* used in this study were grown in open fields located in Zhejiang Province, China. These plants were cultivated using standard agricultural practices, including regular watering, fertilization, and pest control. The geographical coordinates of the cultivation area are approximately 29.18° N latitude and 119.65° E longitude. Three distinct developmental stages of the Dongkui variety were used for transcriptome and metabolic analysis: unripe (UR, 65 days after flowering), mid-ripe (MR, 85 days after flowering), and fully ripe (FR, 100 days after flowering). Three biological replicates were used for the analysis, with a total of ten fruits collected from several randomly chosen plants in each replicate to ensure representativeness.

### 4.2. RNA Sequencing

The total RNA extraction from three stages of *M. rubra* fruit utilized the RNAprep Pure Plant Kit (Tiangen, Beijing, China). RNA quantification involved NanoDrop 2000 (Thermo Scientific™, Waltham, MA, USA) and the RNA Nano 6000 Assay Kit on the Agilent Bioanalyzer 2100 system (Santa Clara, CA, USA). Library preparation for the transcriptome sequencing used the Hieff NGS Ultima Dual-mode mRNA Library Prep Kit for Illumina (San Diego, CA, USA), and the sequencing was performed on an Illumina NovaSeq platform using the PE150 model.

### 4.3. Analysis of RNA Sequencing Data

RNA sequencing data were analyzed as previously described [37]. Briefly, the sequencing reads underwent trimming and alignment to the reference genome of Zaojia using fastp v0.23.2 [38] and HISAT2 v2.2.1 [39], respectively. Gene expression levels were quantified using FPKM (fragments per kilobase of transcript per million mapped reads), and the differential expression analysis employed DESeq2, applying cutoff criteria of an FPKM fold change >2 and a *p*-value of <0.05 [40] (Pertea et al., 2016).

### 4.4. Metabolite Extraction

Biological samples were subjected to vacuum freeze-drying using a Scientz-100F freeze dryer. The dried samples were then ground into a powder with an MM 400 grinder (Retsch, Haan, Germany) at 30 Hz for 1.5 min. Subsequently, 100 mg of the powdered sample was dissolved in 1.2 mL of 70% methanol extraction solution. The mixture was vortexed for 30 s every 30 min, repeating this process six times, and then stored overnight at 4 °C. Following centrifugation at 12,000 rpm for 10 min, the supernatant was extracted, filtered through a 0.22 μm microporous membrane, and stored in sample vials for UPLC-MS/MS analysis.

### 4.5. LC-MS/MS Analysis

The metabolomics analysis from three stages of *M. rubra* fruit utilized the Waters Acquity I-Class PLUS ultra-high performance liquid chromatography tandem Waters Xevo G2-XS QTof high-resolution mass spectrometer (Waters, Milford, CT, USA). The column used was the Waters Acquity UPLC HSS T3 column (1.8 µm 2.1 × 100 mm). In positive ion mode, mobile phase A consisted of a 0.1% formic acid aqueous solution, and mobile phase B was a 0.1% formic acid acetonitrile solution. In negative ion mode, the mobile phases remained the same. The injection volume was set at 1 μL. The Waters Xevo G2-XS QTOF high-resolution mass spectrometer conducted a primary and secondary mass spectrometry data gathering in MSe mode, controlled by MassLynx V4.2 acquisition software. The dual-channel data acquisition involved low and high collision energies in each cycle, with the low collision energy set at 2 V, high collision energy ranging from 10 to 40 V, and a scanning frequency of 0.2 s per mass spectrum. The ESI ion source parameters included capillary voltage (2000 V or −1500 V), cone voltage (30 V), ion source temperature (150 °C), desolvent gas temperature (500 °C), backflush gas flow rate (50 L/h), and desolventizing gas flow rate (800 L/h).

### 4.6. Data Preprocessing and Annotation

Raw data collected using MassLynx V4.2 underwent processing with Progenesis QI software V4 for peak extraction, alignment, and other operations. Progenesis QI used the METLIN database and self-built library (Biomarker Technologies Co., Ltd., Beijing, China) for identification. Theoretical fragment identification and mass deviation were maintained within 100 ppm.

### 4.7. Metabolome Analysis

After normalizing the original peak area information with the total peak area, a principal component analysis (PCA) and Spearman correlation analysis were conducted to assess sample repeatability within groups and quality control samples. Identified compounds were classified, and pathways were determined using the Kyoto Encyclopedia of Genes and Genomes (KEGG) and LipidMaps databases. Based on grouping information, differences were calculated and compared using T tests for significance (*p*-value). The R V4.2 package ropls was employed for OPLS-DA modeling, with 200 permutation tests to verify model reliability. Variable Importance in Projection (VIP) values were calculated through multiple cross-validation. Differential metabolites were screened using a combination of fold change (FC > 2), *p*-value (<0.01), and VIP (>1). The KEGG pathway enrichment significance of differential metabolites was determined using a hypergeometric distribution test.

### 4.8. Quantitative Real-Time PCR

The total RNA from fruits at different stages was extracted using the Trizol method. cDNA was synthesized in a 10 μL reaction using the PrimeScript™ RT Reagent Kit with gDNA Eraser (Takara, Japan). The following components were included in the SYBR Green reaction mix (Takara, Japan): 5 μL of 2× TB Green^®^ Premix Ex Taq™ II FAST qPCR, 0.2 μL each of forward and reverse primers (10 μM), 0.5 μL of cDNA template, and 4.1 μL of ddH_2_O. The reaction was performed on a LightCycler 96 real-time PCR instrument (Roche, Basel, Switzerland) under the following conditions: 95 °C for 60 s, followed by 40 cycles of 95 °C for 15 s, 55 °C for 15 s, and 72 °C for 45 s. Each sample was analyzed, with three technical replicates. Quantitative primers were designed based on gene sequences, with *MrActin* used as the spike-in gene. The data were processed using the 2^−∆∆Ct^ method, and the statistical analysis and plotting were conducted using Origin 2022 and Microsoft Excel 2010, respectively. All primers were designed using multiPrime [27], and the primer sequences are listed in Supplementary Table S1.

## 5. Conclusions

In conclusion, our study provides comprehensive insights into the molecular mechanisms governing fruit ripening in *M. rubra*. The identified genes and metabolites offer potential targets for breeding strategies aimed at enhancing fruit quality and minimizing post-harvest losses. Moreover, our findings contribute to a deeper understanding of the ripening process in this economically important fruit species, laying the foundation for future research endeavors in fruit biology and horticulture.

## Data Availability

The raw sequencing data presented in this paper have been deposited in the Genome Sequence Archive (Accession No. CRA016076) at the National Genomics Data Center, China National Center for Bioinformation/Beijing Institute of Genomics, Chinese Academy of Sciences, and are publicly available at https://ngdc.cncb.ac.cn/gsa. All the data is public and can be accessed at any time.

## Supplementary Materials

The following supporting information can be downloaded at: https://www.mdpi.com/article/10.3390/ijms25168654/s1.
