# Supplementary material for: Transcription-Associated Metabolomic Analysis Reveals the Mechanism of Fruit Ripening during the Development of Chinese Bayberry
Source: Int J Mol Sci. 2024 Aug 8;25(16):8654. doi: 10.3390/ijms25168654 (PMC11355050; doi:10.3390/ijms25168654)
Supplement: Supplementary file 1 [file ijms-25-08654-s001.zip › Supplementary information.pdf]

Transcription-Associated Metabolomic Analysis Reveals the Mechanism of Fruit Ripening during the Development of
Chinese Bayberry

Li Sun [1], Shuwen Zhang [1], Zheping Yu [1], Xiliang Zheng [1], Senmiao Liang [1], Haiying Ren [1] and Xingjiang Qi [1,2,*]

[1] Institute of Horticulture, State Key Laboratory for Managing Biotic and Chemical Threats to Quality and Safety of Agro-Products, Zhejiang Academy of Agricultural Sciences, Hangzhou 310021, China
[2] Xianghu Laboratory, Hangzhou 311231, China
* Correspondence: qixj@zaas.ac.cn

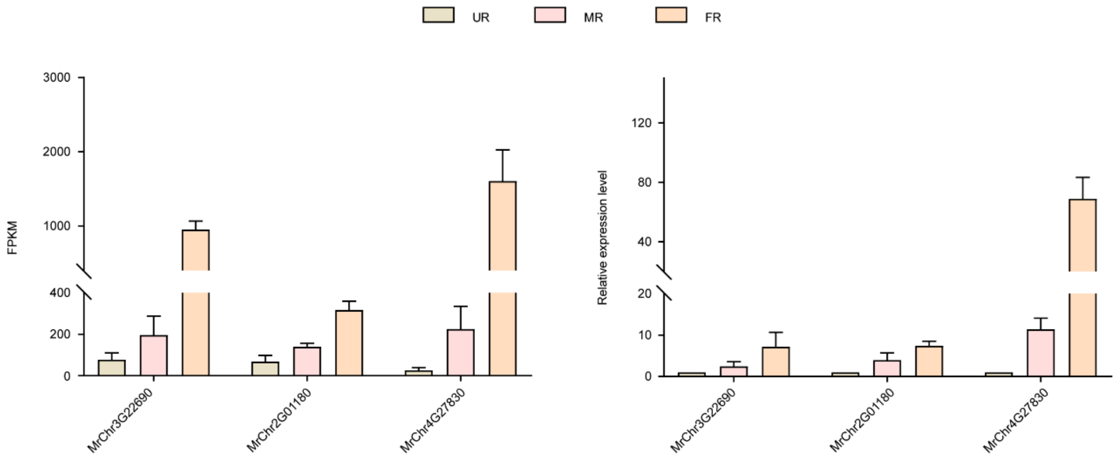

Supplementary Figure S1. Relative abundance of the key genes. A, FPKM values of the key genes. B, Relative expression level (qPCR) of the key genes

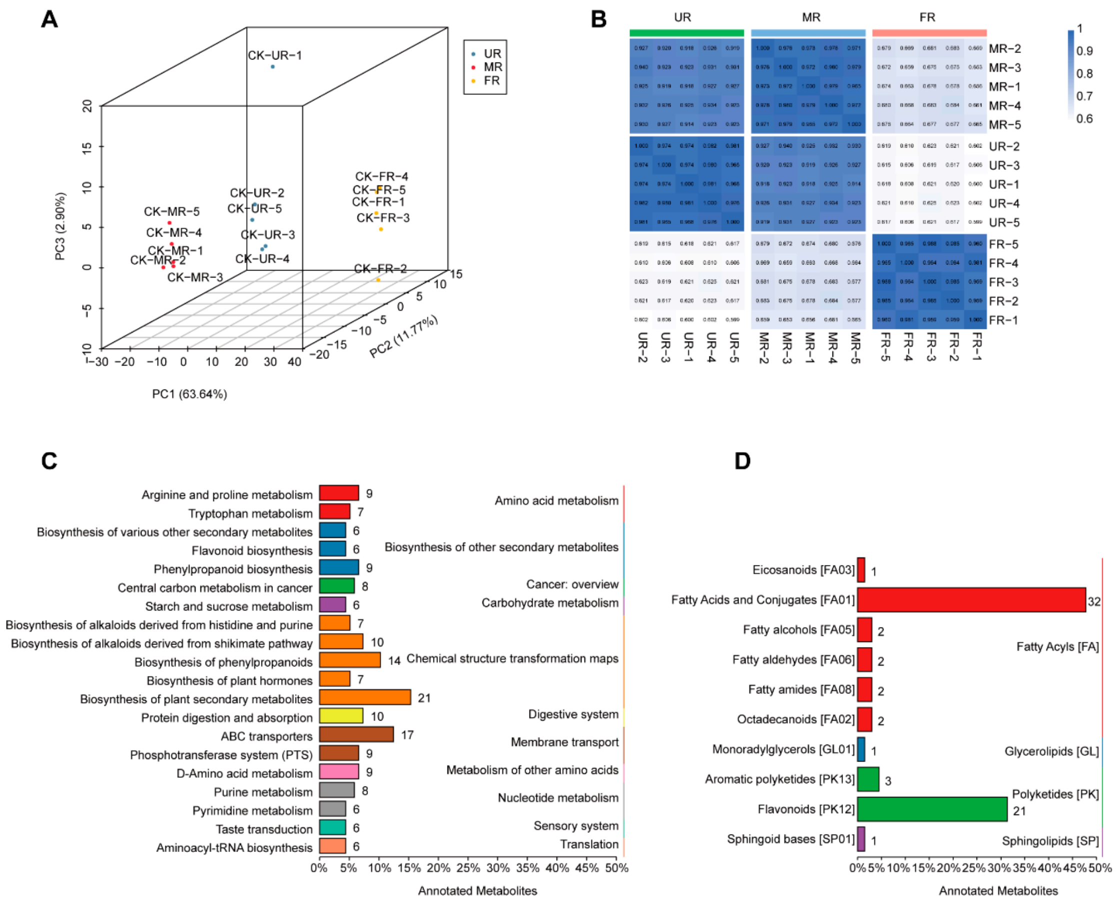

Supplementary Figure S2. Metabolomic correlation and annotation of identified metabolites.

A. PCA plot showing the distribution of samples from the three stages (unripe, middle ripe, and full ripe) of *M.rubra*. **B.** Correlation analysis heatmap depicting the correlation between samples from the three stages of *M.rubra*. **C.** KEGG annotation of the identified metabolites. **D.** Lipidmaps annotation of the identified metabolites.

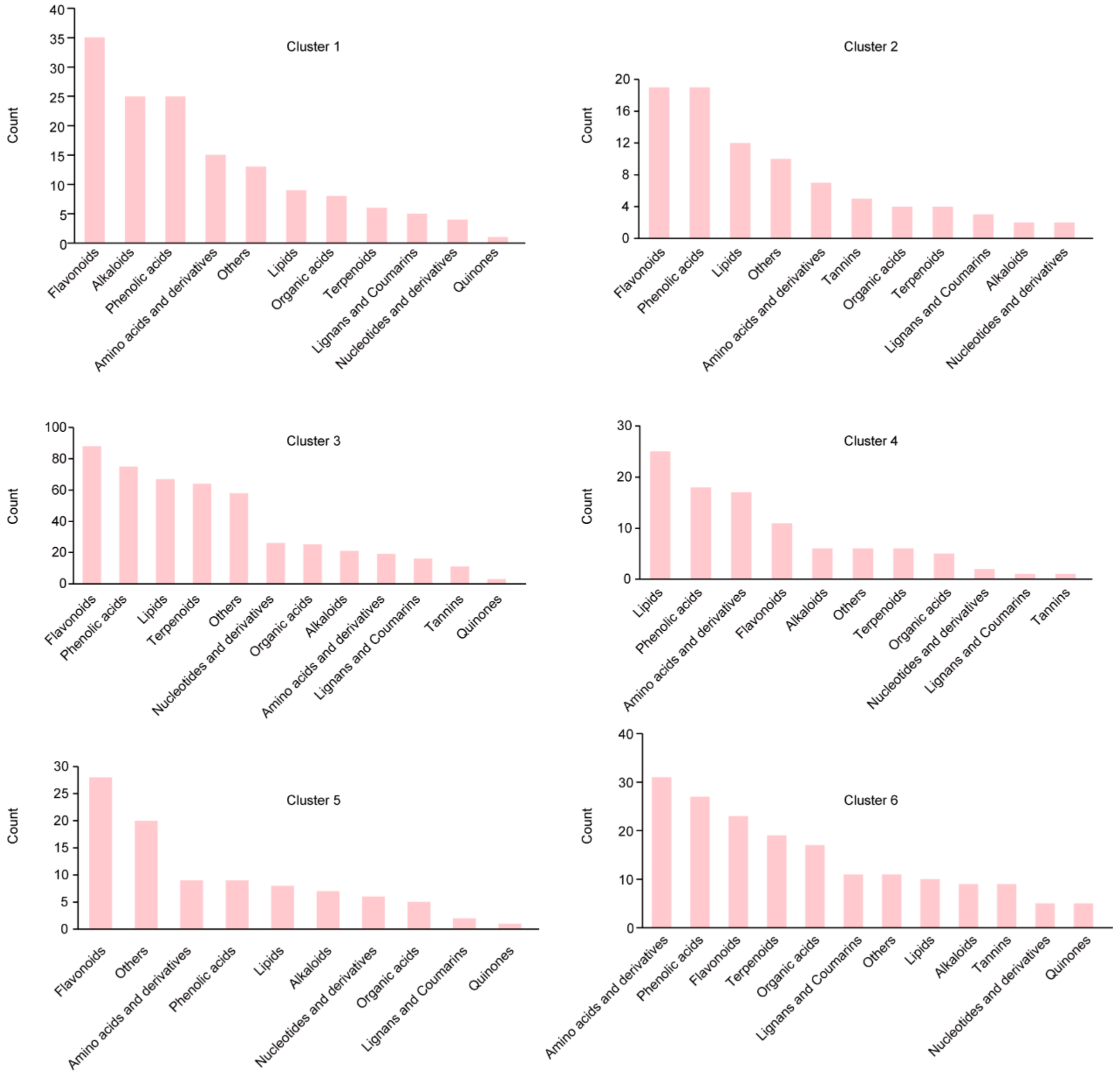

Supplementary Figure S3. Dominant metabolites in each cluster.

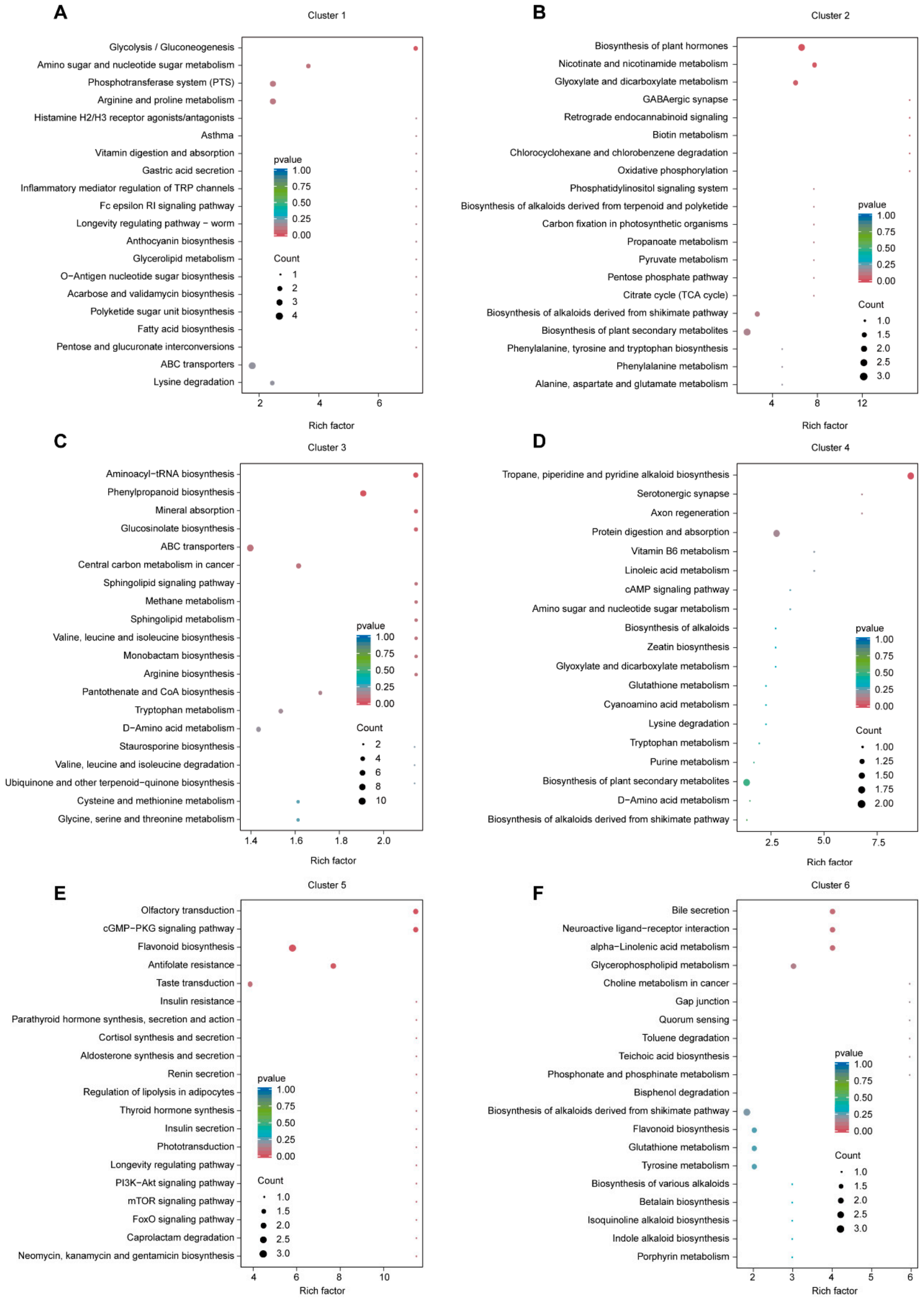

Supplementary Figure S4. GO enrichment analysis of metabolites in time-course clusters

A-F. Cluster 1-6.

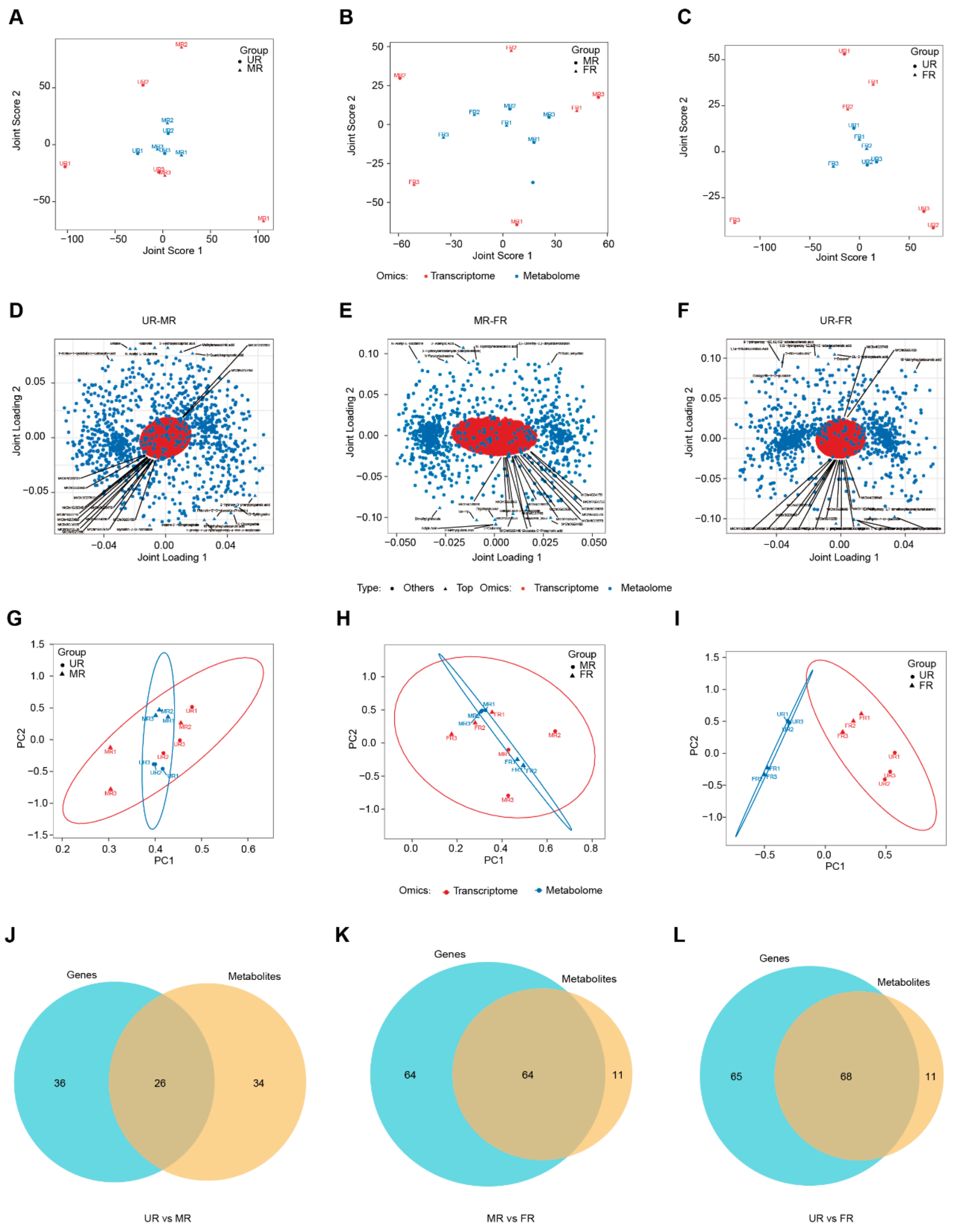

Supplementary Figure S5. O2PLS integration of *M. rubra* metabolites and transcripts. A-C. O2PLS joint scores plot of comparisons between stages (UR-MR, MR-FR, and UR-FR). The shapes of the points (circles and triangles) represent samples from different groups, while the colors of the points represent different omics data. Red denotes transcriptome samples, and blue represents metabolome samples. The horizontal and vertical axes indicate the joint scores of the metabolome and transcriptome. **D-F.** O2PLS loadings plot of transcriptome and

metabolome. The colors of the points in the plot represent different omics data: red denotes differential genes, while blue denotes differential metabolites. The horizontal and vertical axes represent the joint loading values. **G-I.** PCA of the joint analysis. Different coordinates represent different principal components, with the horizontal axis representing the first principal component and the vertical axis representing the second principal component. The shapes of the points (circles and triangles) in the plot represent samples from different groups, while the colors of the points represent different types of omics samples. Red indicates transcriptomic samples, and blue represents metabolomic samples. J-K. The intersection of genes and metabolites across stage comparisons (UR-MR, MR-FR, and UR-FR)
